# Toll‐like receptors in pathogenesis of neurodegenerative diseases and their therapeutic potential

**DOI:** 10.1002/iid3.839

**Published:** 2023-04-19

**Authors:** Yosef Tsegaye Dabi, Abayomi O. Ajagbe, Sisay T. Degechisa

**Affiliations:** ^1^ Department of Medical Laboratory Science, Institute of Health Sciences Wollega University Nekemte Ethiopia; ^2^ Department of Anatomy, College of Health Sciences, Faculty of Basic Medical Sciences Nile University of Nigeria Abuja Nigeria; ^3^ Department of Medical Biochemistry, School of Medicine, College of Health Sciences Addis Ababa University Addis Ababa Ethiopia; ^4^ Department of Medical Laboratory Sciences, College of Medicine and Health Sciences Arba Minch University Arba Minch Ethiopia

**Keywords:** inflammation, neurodegenerative diseases, toll‐like receptors

## Abstract

Toll‐like receptors (TLRs) are a family of pattern‐recognition receptors triggered by pathogen‐derived and tissue‐damage‐related ligands. TLRs were previously believed to only be expressed in immune cells. However, it is now confirmed that they are ubiquitously expressed in cells within the body including neurons, astrocytes, and microglia of the central nervous system (CNS). Activation of TLRs is capable of inducing immunologic and inflammatory responses to injury or infection of CNS. This response is self‐limiting that usually resolves once the infection has been eradicated or the tissue damage has been repaired. However, the persistence of inflammation‐inducing insults or a failure in normal resolution mechanisms may result in overwhelming inflammation which may induce neurodegeneration. This implies that TLRs may play a role in mediating the link between inflammation and neurodegenerative diseases namely Alzheimer's disease, Parkinson's disease, Huntington's disease, stroke, and amyotrophic lateral sclerosis. So, new therapeutic approaches that specifically target TLRs may be developed by better understanding TLR expression mechanisms in the CNS and their connections to particular neurodegenerative disorders. Therefore, this review paper discussed the role of TLRs in neurodegenerative diseases.

## 1 INTRODUCTION

Mammalian cells identify the manifestation of pathogens via a group of receptors complexes known as pattern recognition receptors (PRRs) that are particularized in distinguishing conserved molecular structures essential to the life cycle of a pathogen, and these pathogen‐associated molecular structures are also called pathogen‐associated molecular patterns (PAMPs).^1^, ^2^

Various significant PRRs of the innate immune system are Toll‐like receptors (TLRs), Rig like receptors, Nod‐like receptors (NLRs), and the newly recognized Aim2‐like receptors, C‐type lectin receptors, Dectin 1 or C‐type lectin domain family 7 member A (CLEC7A), dectin 2 or C‐type lectin domain family 6 member A (CLEC6A), DC‐specific ICAM3‐grabbing non‐integrin (DC‐SIGN), and complement receptor 3.^3^, ^4^, ^5^

TLRs are the foremost identified and best‐distinguished family of PRRs regarding their well‐known ligands, downstream signaling channels, and functional significance that assist in the identification of conserved structural motifs in a broad assemblage of pathogens.^6^ TLRs are extraordinarily preserved PRRs that are available in animals as low as nematodes, namely *Caenorhabditis elegans*.^7^

Preliminary uncovering of the mammalian TLRs occurred due to their sequence resemblances to Toll, a receptor located in Drosophila dorsoventral embryonic evolution and antifungal immunity.^8^ TLRs play role in the stimulation of the inborn immune system.^9^, ^10^, ^11^ PAMPs namely lipopolysaccharide (LPS), and damage‐associated molecular patterns (DAMPs) viz high‐mobility group box 1 (HMGB1) are recognized by TLRs.^10^, ^11^, ^12^, ^13^, ^14^

Hitherto, 13 TLRs have been recognized in mice and 11 in human tissues and cells with distinct cellular distribution and placement namely in the immune system for example dendritic cells (DC), macrophages, neutrophils, and B and T cells and the nonimmune system namely fibroblasts, epithelial cells, and myocardia and also in neurons and glial cells of both peripheral nervous system and the central nervous system (CNS).^14^, ^15^ TLR1, TLR2, TLR4, TLR5, TLR6, and TLR10 are exhibited on the cell surface and have been disclosed to recognize and cause a response to extracellular bacterial‐related ligands while TLR3, TLR7, TLR8, and TLR9 are situated in the endosomes of specific immune system cells.^9^, ^10^, ^15^

TLR‐mediated neuroinflammation has been linked to a wide range of infectious and noninfectious neurological and neurodegenerative CNS conditions.^2^, ^16^, ^17^ TLRs have also been shown to influence neural development and cognitive function.^16^ This review paper summarized findings demonstrating the function of TLRs in neurodegenerative diseases.

## 2 TLRS

### 2.1 Structure and function of TLRs

TLRs are evolutionarily conserved receptors in the PRR family. PRRs are transmembrane proteins that play a crucial role in immune responses by inducing signals in response to PAMPs.^5^, ^18^ TLRs are distinguished by N‐terminal leucine‐rich repeats (LRRs), a transmembrane region, and a cytoplasmic Toll/IL‐1R homology (TIR) domain. Different TLRs identify different molecular patterns of microbes and self‐components.^18^

TLR transmembrane domains typically comprise a length of about 20 uncharged, mainly hydrophobic residues. TLR N‐terminal ectodomains (ECDs) are glycoproteins containing 550−800 amino acid residues. These ECDs can be found either extracellularly or in endosomes, where they interact with and identify molecules produced by invading pathogens.^6^

The horseshoe‐shaped structure of the N‐terminal LRRs mediates the recognition of foreign and endogenous pattern molecules. The TIR domain connects to adapter molecules and triggers signal transduction. TLR‐dependent recognition of PAMPs activates the innate immune system, which in turn activates antigen‐specific adaptive immunity.^19^ TLRs are widely known for their role in the recognition of microbes. Each TLR recognizes distinct microbial component patterns.

### 2.2 TLR signaling

Each immune cell possesses a distinct group of TLRs that identify and interact with PAMPs, resulting in the generation of proinflammatory cytokines and type 1 interferon, which directs immune responses to specific microorganisms. TLR ligand binding activates various intracellular downstream signaling cascades that induce host defensive responses.^20^ Since TLRs are transmembrane receptors of class I, ligand binding induces dimerization, which activates them. The type of stimulus, the activated TLR, and the downstream adapter molecule all influence TLR signaling. TLR signaling involves at least two distinct pathways: (i) the Myeloid differentiation primary response 88 (MyD88)‐dependent pathway, which is used by all TLRs except TLR3 and leads to the production of inflammatory cytokines; and (ii) the TIR‐domain‐containing adapter‐inducing interferon‐β (TRIF)‐dependent pathway is also called MyD88 independent pathway, which is used by TLR3 and 4 and is associated with the activation of interferon type‐1.^21^, ^22^

Upon activation by PAMPs or DAMPs, TLRs recruit adapter proteins. The recruitment of MyD88 to the TIR domain of TLRs induces IL‐1R‐associated protein kinases (IRAK) 4 and IRAK1/2 binding to form the Myddosome. Activation of tumor necrosis factor (TNF) receptor‐associated factor (TRAF) 6 downstream of MyD88 leads to the activation of nuclear factor kappa‐B (NF‐κB), IFN regulatory factors (IRF) 5, and the Mitogen‐activated protein kinases (MAPK) pathways, inducing the expression of proinflammatory cytokines. The association of TRIF with TLR3 and internalized TLR4 also leads to the expression of proinflammatory cytokines via TRAF6 and receptor‐interacting serine/threonine‐protein kinase (RIPK) 1. TRAF3 also mediates the expression of type I IFNs following activation of the IRFs.^22^

TLR4 has a distinguishing feature from all other TLRs in that its activation requires myeloid differentiation factor 2 (MD‐2) which is an accessory protein and in its activated phase, TLR4 attracts pair of distinct downstream signaling operations; MyD88‐dependent and MyD88‐independent routes.^11^

The adapter protein TIRAP is the requisite for MyD88‐dependent pathway in TLR4 signaling and for MyD88 to launch a downstream cascade resulting in nuclear translocation of the NF‐κB and MAPK signaling pathways (such as the extracellular signal‐regulated kinases‐cyclic adenosine 3′,5′‐monophosphate [cAMP] response element [CRE] binding protein [ERK‐CREB] pathway, the Janus kinase‐Activator protein 1 [JNK‐AP1] pathway, and the p38 pathways), leading to the production of proinflammatory cytokines.^23^

This cause the swift exhibition of inducible nitric oxide synthase (iNOS) and a vast diversity of proinflammatory cytokines, chemokines, and their receptors, namely TNF‐alpha (TNF‐α), IL‐1α, IL‐1β, IL‐1ra, IL‐6, IL‐8, IL‐10, IL‐12p40, IL‐23, and macrophage inflammatory protein (MIP)‐1α, and MIP‐1β. These factors start the inflammatory response, elevate vascular permeability, direct DC, and macrophage migration from the periphery to the central lymphoid organs, and regulate various aspects of adaptive immunity development.^23^

TLR activation is characterized by cytokines, which are short‐lived proteins that act on many tissues to generate a systemic response, making them the primary contributors to behavioral changes during an infection such as weakness, lethargy, weariness, and anorexia.^24^ They can also have an impact on physiological systems by directing metabolic processes toward catabolism, inhibiting muscle protein synthesis, increasing muscle atrophy, and causing hepatic anabolism. They also stimulate peripheral lipolysis, enhance de novo fatty acid synthesis and hepatic triglyceride formation, and boost VLDL‐cholesterol secretion, all of which raise blood triglyceride levels.^25^

### 2.3 Cooperation between TLRs and Node‐like receptor protein 3 (NLRP3) inflammasome

Multiple types of brain cells have been shown to contain specialized PRRs, including membrane‐bound TLRs and cytosolic NLRs. One of the most extensively studied classes of NLRs is the inflammasome‐forming NLRs of which NLRP3 inflammasome is the best characterized.^26^ TLRs and NLRP3 inflammasome cooperation is a well‐known phenomenon which results in IL‐1 and IL‐18 secretion.^27^ The NLRP3 inflammasome is assembled from NLRP3, an apoptosis‐associated speck‐like protein containing a caspase activation and recruitment domain (ASC) adapter protein, and the downstream effector enzyme (procaspase‐1).^27^, ^28^, ^29^ When stimulated by PAMPs or DAMPs, the formation of NLRP3 inflammasome is actualized. This initiates the cleavage of procaspase‐1 into the active and mature form of caspase‐1. Activated procaspase 1 in turn activates the inactive pro‐IL‐1 and pro‐IL‐18 are then changed into their active and secreted counterparts, IL‐1 and IL‐18. These cytokines trigger proinflammatory responses and several downstream signaling pathways, which can result in cellular destruction such as autophagy and pyroptosis.^29^ Therefore, NLRP3 signaling is required for transcriptional induction of pro‐IL‐1 and pro‐IL‐18, followed by proteolytic processing via inflammasome activation as extensively reviewed elsewhere.^29^, ^30^, ^31^

## 3 TLRS IN THE NERVOUS SYSTEM

### 3.1 Expression of TLRs

In the nervous system, the microglial cells principally emerge to express a wide span of TLRs and as such are known to play a protective role in the brain.^32^, ^33^ TLRs are widely expressed in astrocytes and oligodendrocytes, where the main receptors are TLR2 and TLR3, and TLR2 is one of the most abundant TLRs in the CNS.^32^, ^33^, ^34^, ^35^, ^36^

Prior research has shown that neurons do not express TLRs in any way; nevertheless, Tang et al. found TLR2, TLR4, TLR5, and TLR9 in cortical neurons.^35^ By employing multiple technologies, it was demonstrated that primary mouse cortical neurons exhibit TLR2, TLR3, and TRL4. Primarily, single‐cell molecular analysis was applied to demonstrate the availability of TLR2 and TLR4 messenger ribonucleic acid in neurons.^32^, ^35^, ^36^

Investigating the expression of TLR1‐9 in neuronal cultures via the application of microarray analysis disclosed that TLR2, TLR3, TLR6, TLR7, and TLR8 were exhibited at approximately low degrees, TLR2 and TLR4 at intermediate degrees, and TLR5 and TLR9 at higher degrees.^35^ Furthermore, TLRs are expressed in leukocytes where their role has been confirmed and the latest studies propound they can also be indicated in nonimmune cells like hepatocytes and muscle cells.^35^

### 3.2 TLRS in CNS

Neurogenesis takes place not just in the developing brain but also in the mature brain of mammals. The adult brain encompasses a complex activity that includes proliferation, differentiation, migration, and integration of neural stem cells (NSCs) into a neural network.^36^ It has been established that NSCs exist principally in pair areas of the adult brain namely the subgranular zone (SGZ) of the hippocampal dentate gyrus (DG) and the subventricular zone (SVZ) of the lateral ventricles.^36^ Adult hippocampal neurogenesis is dependent both on innate neural progenitor cells (NPC‐derived) and external (adjacent endothelial cells, astrocytes, and microglia) signals which demonstrate TLRs.^34^

TLR2 is one of the major predominant TLRs in the CNS and its immunoreactivity investigation in the adult brain neurogenic areas disclosed its expression on cells in both the SGZ of the hippocampal DG and the SVZ of the lateral ventricles identified for neurogenesis.^37^ Furthermore, studies have revealed that TLR2 can be found in a variety of brain cells, including neurons, microglia, oligodendrocytes, and astrocytes.^36^

To verify the expression of TLR2 in the adult neurogenic regions, a study was conducted to juxtapose amounts of proliferating cells in slices acquired from the DG of TLR2‐deficient (TLR2D) mice and wild‐type C57BL/6 (control mice) after the administration of cell‐proliferation marker 5‐bromodeoxyuridine (BrdU).^34^, ^36^ Investigation of whether TLR2 insufficiency influences the differentiation of neural stem/progenitor cells (NPCs) into neurons revealed a remarkably smaller degree of BrdU+ cells coexpressing Doublecortin (DCX) in TLR2D mice than in the wild‐type and a homogenous depletion in novel neurons was spotted by staining of the neuronal marker, β‐III tubulin (βIIIT).^34^

To investigate by what means the TLR2 exhibited on NPCs influences the differentiation into neurons, pharmacological activators of TLR2 (the lipopeptide Pam3CysSK_4_ [PSC] and peptidoglycan [PG, a cell‐wall constituent of Gram‐positive organisms]) and TLR2‐neutralizing antibodies were introduced.^34^ Introduction of the TLR2 activators to the wild‐type NPCs remarkably increased their differentiation, exhibited in a dose‐dependent surge in the degree of cells demonstrating the neuronal marker βӀӀӀT and a change in their framework.^34^

## 4 TLRS IN NEURODEGENERATIVE DISEASES

There is a relationship between the activity of TLRs and various neurodegenerative diseases such as stroke, amyotrophic lateral sclerosis (ALS), Parkinson's disease (PD), and Alzheimer's disease (AD) as TLRs are exhibited in diverse cell types namely neurons and glia where they discern DAMPs released by undifferentiated or necrotic cells.^10^, ^38^

Taking TLR4 as an example, the recognition of LPS by TLR4 entails the binding of circulating LPS to LBP (lipid‐binding protein) and migrates LPS to CD14 (glycophosphatidylinositol‐anchored membrane protein) that exists in the soluble form and holds together LPS‐LBP complex and is accountable for the identification of smooth LPS. Membrane‐bound CD14 lack its intracellular domain, amalgamates, and joins with TLR4 to evolve a functional LPS receptor complex.^7^

MD‐2 molecule is essential for the binding of LPS to TLR4 as it relates itself to the extracellular domain of TLR4. Therefore, the functional LPS network is made up of TLR4, CD14, and MD‐2 where CD14 and LBP are in charge of boosting the TLR4‐dependent LPS signaling and cell activation. Immediately the extracellular binding of LPS to TLR4 is achieved, it causes the downstream signaling consisting of diverse adapter molecules, kinases, phosphatases, and resulting stimulation of NF‐κB resulting in the generation of proinflammatory cytokines (TNF‐α, IL‐1α, IL‐6, IL‐8) plus other molecules accountable for the integration of inflammatory routes in charge of neuroinflammation.^7^

### 4.1 TLRs in AD

AD is the most frequent age‐related neurodegenerative disorder. The most common pathophysiology of AD is characterized by extensive deposits of Amyloid beta (Aβ) plaques in the extracellular space and intracellular accumulation of neurofibrillary tangles.^39^, ^40^, ^41^

In the case of AD, TLR4 is assumed to act as a mediator for the neurotoxic activities of DAMPs related to neuronal injury involved in AD. The study has disclosed that a prototypic DAMP named HMGB1 released from necrotic or hyper excitatory neurons affects neurite debasement through TLR4.^10^ The mechanism of action entails the binding of HMGB1 released from necrotic or hyper excitatory neurons with TLR4 and results in the activation of MAP kinases affecting myristoylated alanine‐rich C‐kinase substrate (MARCKS) phosphorylation resulting in the deterioration of neurites which is a fundamental landmark of AD pathology.^10^ MARCKS is a submembrane protein required in the actin network stability and is phosphorylated at ser46 well before accumulation of the Aβ.^10^

### 4.2 TLRs in PD

PD is a neurodegenerative condition marked by tremors, unbalanced gait, rigidity, and non‐motor deficits. Although the precise cause of intermittent PD is unknown, mounting evidence suggests that misfolded α‐synuclein activates microglial cells in the substantia nigra pars compacta (SNc), causing them to become more inflamed and under oxidative stress, which in turn causes neurodegeneration.^14^

TLR2 is the most studied neuronal TLR in the context of PD pathology. TLR2 expression is increased in PD, according to an immunocytochemical analysis of postmortem human brains.^42^ TLR2 was exclusively expressed in microglia in control brains, but in affected individuals, TLR2 expression was predominantly upregulated in neurons of the substantia nigra and anterior cingulate cortex. Extracellular misfolded, fibrillar α synuclein produced from neural cells is identified as PAMP by microglia TLR2 which in turn triggers downstream channels entailing MyD88 and NF‐κB activating the creation of TNF and 1l‐1β.^14^ Furthermore, there was a strong correlation between an increase in neuronal TLR2 expression and disease stage.^42^ These findings suggested that increased neuronal TLR2 expression may contribute to the pathogenesis and progression of PD.

TLR2 deficient α‐synuclein A53T mice were created to investigate the relationship between TLR2 signaling and α‐syn pathology in neurons. TLR2 deficiency reduced α‐syn accumulation in neurons, reduced neuronal loss, and improved motor deficits. In addition to these in vivo findings, the same study found that Pam3CSK4‐induced TLR2 activation results in α‐syn accumulation in a human neuroblastoma cell line overexpressing α‐syn. This was due to impaired autophagy, as activating autophagy decreased α‐syn accumulation in response to Pam3CSK4, whereas inhibiting the autophagy/lysosome degradation pathways promoted α‐syn accumulation in these cells. The TLR2‐dependent activation of the AKT/mTOR signaling pathway inhibited autophagy.^43^, ^44^

In in vitro studies, TLR4 disclose to communicate with alpha‐synuclein, activating the response of microglia namely alpha‐synuclein uptake, proinflammatory cytokine release and oxidative stress stimulation and these findings were supported by a 1‐methyl‐4‐phenyl‐1,2,3,6 tetrahydropyridin (MPTP) induced murine prototype of PD where the genetic nonappearance of TLR4 signaling safeguarded the mice from neurodegeneration disclosing the chief function of TLR4 in the development of PD.^14^

### 4.3 TLRs in ALS

ALS is a neurodegenerative disease that develops in adults and causes selective loss of upper and lower motor neurons in the brain and spinal cord, resulting in paralysis and death.^45^ ALS is the most rapidly fatal of the neurodegenerative disorders, typically taking 2−3 years from symptom onset to death.^46^, ^47^

Most ALS cases are sporadic, but about 15% are familial with Mendelian inheritance patterns and high penetrance.^48^, ^49^ Numerous genes have been linked to familial ALS mutations, with superoxide dismutase 1 (SOD1) being the most thoroughly researched.^50^

It is generally accepted that glutamate‐induced excitotoxicity, abnormal mitochondrial structure, autophagy, neuroinflammation, and disruption of axonal transport mechanisms form the pathophysiological basis of ALS.^51^ Astrocytes and microglia are non‐neuronal cells that contribute to ALS‐related neurodegeneration by secreting neurotoxic agents and altering the expression of glutamate receptors.^51^ TLR2 and TLR4 transcript and protein levels were found to have increased globally in the spinal cord after analysis of postmortem tissue taken from sporadic ALS patients.^52^

According to immunocytochemical colocalization studies, TLR2 is primarily expressed by microglia/macrophages, whereas TLR4 is expressed by Glial fibrillary acidic protein‐positive astrocytes in the white and gray matter of the cervical spinal cord obtained from ALS patients. In the glia of control tissue, neither TLR2 nor TLR4 are expressed.^52^

Studies on mice that express human mutant SOD1 further supported the idea that glial TLR4 is involved in ALS‐like disease. Control mice exhibit TLR4 expression in their motor neurons but not in their glia. Nevertheless, at later stages of the illness, TLR4 expression is seen in both reactive astrocytes and activated microglia of mutant hSOD1 mice. Genetic deletion of TLR4 improves neurological deficits and increases survival in mutant hSOD1 mice.^53^ These studies provide evidence that increased glial TLR4 signaling plays a role in disease pathology.

### 4.4 TLRs in multiple sclerosis (MS)

Complex gene‐environment interactions are involved in the development of MS, despite the exact cause and the mechanisms underlying its rise being unknown.^54^ TLRs and their signaling proteins are thought to play a significant role in the pathogenesis of MS. It has been discovered that TLRs are expressed in the glial cells of MS patients' CNS.^55^ In neuroinflammatory models, TLR2, TLR9, MyD88, and IRF‐3 deficiency resulted in protective effects, whereas TLR4, TLR2, and TRIF deficiency resulted in aggravating disease, indicating the complex role of TLRs in inflammatory development in MS.^56^, ^57^, ^58^, ^59^, ^60^, ^61^

### 4.5 TLRs in cerebral ischemia/stroke

According to neuroscientists, microglial activation is the primary cause of inflammation following cerebral ischemia, and TLR members have a significant influence on this activation. Blood flow is eventually reduced during a stroke, resulting in several conditions such as ionic imbalance, acidosis, and excitotoxicity due to a lack of oxygen and glucose. Damage to cellular constituents and the release of DAMPS that activate specific TLRs occur sequentially.^62^, ^63^

TLR2 expression in the brain is increased in rodent models of transient and permanent focal cerebral ischemia, as well as in vitro ischemia. TLR2‐related genes with proinflammatory and proapoptotic properties were activated following ischemia. TLR2, TLR4, NF‐κB, cyclooxygenase‐2 (COX‐2), and serum TNF‐α were found to be correlated with ischemia‐induced cerebral inflammation and brain injury in rats. TLR4 expression in microglia in the brain is also increased in the transient cerebral ischemia mouse model.^64^, ^65^, ^66^

### 4.6 TLRs in Huntington's disease (HD)

HD is a neurodegenerative illness that affects the brain and causes gradual loss of motor, cognitive, and psychiatric functions. A mutation in the Huntingtin (HTT) gene causes the expression of an aberrant protein that accumulates in the brain and damages neurons.^67^

Neuroinflammation is believed to be involved in the neurodegenerative process of HD. The abnormal protein produced by the mutated Huntingtin gene in HD has been shown to triggers an immune response in the brain, leading to chronic inflammation. This chronic inflammation can damage neurons and contribute to the progressive loss of brain function seen in HD.^68^ Inflammatory molecules such as cytokines, chemokines, and reactive oxygen species (ROS) have been found to be elevated in the brains of individuals with HD. These molecules can activate microglia, the immune cells in the brain, leading to further inflammation and damage to neurons.^67^, ^68^

Activation of TLR4 trigger downstream signaling pathways in the glia, inducing secretion of ROS and cytokines which result in the degeneration where chronic activation of TLR4 is believed to be related to glia‐mediated neuronal death as a result of unrestricted secretion of cytotoxins.^11^

## 5 THERAPEUTIC APPROACHES

TLR modulation has emerged as an appealing therapeutic approach because they are an essential part of the innate immune system responsible for the initiation of the immune response, TLRs modulate homeostasis, neuronal morphogenesis, and neurogenesis, and TLRs have been linked to the pathology of neurodegenerative disorders.^9^, ^13^, ^69^, ^70^, ^71^

TLR2 therapeutic targeting reduces Amyloid β1‐42 accumulation in the hippocampus of an AD animal model and alters the progression of memory loss.^61^ In a Parkinson's transgenic mouse model, immunization with human α‐synuclein was associated with a significant reduction in accumulated α‐Synuclein and overall reduced neurodegeneration, indicating that α‐Synuclein vaccination could be effective in reducing neurodegeneration associated with accumulated α‐Synuclein.^72^

In several models of cerebral ischemia, a low dose of the TLR4 ligand LPS administered systemically reduced brain damage. Another potential target for brain ischemic preconditioning has been identified: TLR9. When administered systemically, the specific mouse TLR9 ligand CpG ODN 1826 causes neuronal protection against ensuing cerebral ischemia.^73^, ^74^

Researchers have demonstrated various polyphenol classes, including stilbenes, phenolic acids, flavonoids, and phenolic alcohols may target TLR signaling through a different pathway. Polyphenols' pleiotropic activity, including antioxidant properties, can modulate the important pathogenesis of ND. Polyphenols, for example, can modulate the NF‐κB mediated pathway to provide neuroprotection.^75^

TLRs are important in neuroinflammation and the development of neurodegenerative diseases such as Alzheimer's, Parkinson's, and MS. TLR inhibition may be a chief therapeutic approach for preventing neurodegenerative disorders caused by various inflammatory markers.

## 6 CONCLUSION

Neurodegeneration is a pathological condition marked by the activation of various neuronal inflammatory cytokine and chemokine cascades. Astrocytes are activated by pathogenic proteins that accumulate in neurodegenerative diseases. These proteins may act as endogenous TLR ligands in astrocytes, inducing proinflammatory responses. Other CNS cells may function differently as a result of the chemokines and cytokines released. TLR activation can also protect neurons from toxicity caused by protein aggregates because TLRs facilitate their internalization and clearance by astrocytes.

TLRs are involved in various physiological pathways and pathological conditions, as evidenced by the literature, and studies that specifically delete these receptors in microglia using neurodegeneration models could help clarify their roles in neuropathological conditions. To gain control over inflammation‐induced NDs, it is necessary to understand TLRs' complex signaling in the brain. TLR targeting would be an excellent treatment strategy, with significant implications for the development of novel therapeutics for these diseases.

## AUTHOR CONTRIBUTIONS

**Yosef Tsegaye Dabi**: Conceptualization; data curation; formal analysis; investigation; methodology; resources; software; supervision; validation; visualization; writing—original draft; writing—review & editing. **Abayomi O. Ajagbe**: Data curation; formal analysis; investigation; methodology; resources; software; validation; visualization; writing—original draft; writing—review & editing. **Sisay T. Degechisa**: Conceptualization; data curation; formal analysis; resources; software; supervision; validation; visualization; writing—original draft; writing—review & editing.

## CONFLICT OF INTEREST STATEMENT

The authors declare no conflict of interest.

## Data Availability

Data sharing is not applicable to this article as no data sets were generated or analyzed during the current study.
